# Association between Internet Usage and Quality of Life of Elderly People in England: Evidence from the English Longitudinal Study of Ageing (ELSA)

**DOI:** 10.3390/ijerph192315544

**Published:** 2022-11-23

**Authors:** Agatha Ravi Vidiasratri, Peter A. Bath

**Affiliations:** 1 Public Health, School of Health and Related Research, The University of Sheffield, Sheffield S10 2TN, UK; 2 Department of Preventive and Community Dentistry, Faculty of Dentistry, Universitas Gadjah Mada, Yogyakarta 55281, Indonesia; 3 Information School and School of Health and Related Research, The University of Sheffield, Sheffield S10 2TN, UK

**Keywords:** quality of life, older people, internet

## Abstract

The WHO has stated that the number of senior citizens above age 65 across the world will double by the year 2050: in the UK, the whole population is projected to grow by about 2.5% over a decade, from mid-2018. Although people are living longer, they are not healthier in old age, and there is an increasing number of illnesses and disabilities in the ageing population, which have an impact on their overall well-being and quality of life (QoL). Alongside these trends, Internet technologies have improved and provide a wide range of information, including on medical and health issues. This study aimed to examine the association between the utilisation of the internet among older people in England and their QoL. This study utilised the English Longitudinal Study of Aging (ELSA), a longitudinal study of a representative sample of people aged 50 and over in England. The data from Wave 9 were analysed using bivariate analysis and logistic regression. The results show a strong association between QoL and utilisation of the Internet in older people, even when adjusting for demographic variables and health. Higher use of the internet was associated with older people being less likely to have higher QoL. The excessive use of the internet for communication and gathering information also contributed to lower QoL. From the findings, poorer QoL was also found in people in older age groups, in those who are married, and those who never suffer from chronic diseases. Our findings suggest that the quality of life in older people might not only be associated with the frequency of usage but also the purpose for which the internet is used; however, this relationship is complex and further research should explore this in greater depth. Further research should also investigate how older people’s use of the Internet changed during the COVID-19 pandemic and the effects of this on the QoL in older age.

## 1. Introduction

Ageing within populations is happening across the world. The UK ageing population, i.e., those aged 65 years and above, has grown from 9.1 million people and is predicted to increase to almost 12 million people in the next 25 years, representing an increase from 15.8 to 18 percent of the total population [1]. Over the past half-century, the mortality rates worldwide, and particularly in developed countries, including the UK, have decreased [1]. Declining mortality rates have led to higher life expectancies; however, although people are living longer, they are not necessarily living healthier lives as they age. Based on a survey among older people in England, nearly 30 percent of older citizens in England, aged 60 to 64 years in 2016, were diagnosed with two or more chronic, i.e., long-term, illnesses. Common long-term illnesses among older adults include cardiovascular disease, cancer, hypertension, osteoporosis, and diabetes mellitus [1,2]. Given the increasing number of older people and the increasing prevalence of chronic diseases, both globally and in the UK, greater attention should be paid to understanding the causes and how the conditions can be managed, by both health services and older people themselves.

The existing research suggests that multimorbidity (i.e., the presence of two or more long-term conditions) negatively impacts an older person’s life, leading to higher levels of dependency, reduced productivity and poorer health-related quality of life [3]. Digital and other technologies have significantly improved the management of long-term illness and, more generally, the Internet provides increased access to large amounts of information: a wide array of tasks that can potentially support a better healthy lifestyle and management of a person’s illness [4,5,6,7]. Based on several surveys, internet use among older people grew gradually from 2014 to 2019, at 13 per cent, and by approximately 20 per cent among British people aged over 50 years between 2010 and 2016 [8,9]. 

Several studies have found that older populations having health issues and feeling remote from others can benefit from using technology, e.g., computers and the Internet, due to the Internet’s capacity for users to undertake a variety of tasks [6,7]. The Internet can also potentially foster a healthy lifestyle and support the management of long-term illnesses [5]. In addition, Internet-based technologies are able to provide a more comprehensive social network for older people to reduce social isolation, as well as being a source of information on leisure activities and health-related information [10,11]. However, frequent Internet use may also cause older people to participate in fewer social activities [12], which can adversely affect their QoL. Thus, this study sought to explore the association between Internet use and quality of life among older people in England.

Evidence of the association between internet use and quality of life in older persons has been shown in previous research. A literature review by Damant et al. (2016) reported the positive effects of digital engagement, such as using email or Skype, on older adults’ quality of life, due to its ability to connect senior citizens with their communities [13]. Khalaila and Vitman (2017) conducted a cross-sectional study on people aged 50 and over in northern Israel: using a sample of 525 adults, they demonstrated a correlation between the combination of Internet use and social networks and quality of life [14]. This highlights the value of social connections in reducing loneliness and improving quality of life. However, the study found that the Internet only improves adults’ quality of life if they also have direct contact with their family members. Another study of people aged 50 years and over showed that Internet use positively correlates with their life satisfaction. However, the study did not provide details of what aspects of Internet use they measured. Therefore, it was not clear from this study whether the positive impacts resulted from more social interaction among the older people, or for other reasons [15]. Until now, no research has analysed the strength of the association between Internet use and quality of life among older people in England, including their detailed use of the Internet, such as the intensity and for what purposes it was used. More generally, there has only been limited research on how strong the relationship is between Internet use and QoL, and other sociodemographic and health variables in older people. Because of these gaps in the research literature, our study used the English Longitudinal Study of Ageing (ELSA), which includes a representative sample of older people, to explore the relationship between Internet use and quality of life, and the moderating effects of sociodemographic and health variables.

## 2. Materials and Methods

This research is a cross-sectional study utilising data from Wave 9 (2018–2019) of the English Longitudinal Study of Aging (ELSA): this was the first wave that included questions and variables on Internet use and information on older people’s activity in accessing healthcare information. The ELSA is a national, population-based cohort study, containing a representative sample of people aged 50 and over, who were originally sampled in 2002. The sample has been followed up at two-year intervals, with re-sampling within the population to replace the participants who died or left the study for other reasons. Computer-assisted personal interviews with cognitive and physical examinations and self-reported questionnaires were used for the data collection. Data cleaning was conducted as the first step to prepare the raw data for analysis. Initially, sample members from wave nine (*n* = 8736) were selected, and this was then restricted to only those people for whom all data were available and who met the requirements: this resulted in 5272 individuals available for the analyses.

The demographic and health variables were taken from the datasets, including gender, age, ethnic group, marital status, education, socioeconomic status, and long-standing illness history. The other variables used in this research were the utilisation of the Internet and quality of life.

During Wave 9 of ELSA, the participants were asked questions on 12 different purposes for using the Internet over the past three months. For this study, those purposes were divided into six groups: (1) communication (sending or receiving emails; use of social networking sites; and creating, uploading, or sharing content); (2) entertainment (news, newspaper or blog websites, streaming or downloading live or on-demand television or radio, music, electronic books, and games); (3) information access (finding information about goods and services; searching for information for learning, research, and fact-finding; and looking for a job or sending a job application); (4) finance management; (5) electronic commerce (shopping and buying goods or services over the Internet or selling goods or services over the Internet), and (6) other [16]. These purposes were identified as potentially influencing people’s health condition, whether physically or mentally.

### 2.1. Quality of Life Measurement

Quality of life was measured using the Control, Autonomy, Self-Realization and Pleasure (CASP-19) measures of QoL, which uses 19 Likert scale questions in a self-reported questionnaire [17]. This scale, which is grouped into four domains (shown in Table 1): Control, Autonomy, Pleasure, and Self-Realisation, has been validated for measuring older people’s quality of life [15]. Each CASP-19 item is scored on a scale of ‘Often’ (scored 4), ‘Sometimes’ (scored 3), ‘Not often’ (scored 2), and ‘Never’ (scored 1) and has a score range between 19 and 76, with a high score representing a higher quality of life. The quality-of-life variables were summed, and the median was analysed. The data were then categorised and transformed into two groups: low QoL (0) for those scoring below the median, and high QoL (1) for the individuals scoring the median and above.

### 2.2. Statistical Analysis

The data were cleaned, transformed, and analysed using the Statistical Package for the Social Sciences (SPSS). The means, standard deviation (SD), and percentage of the essential parameters by frequency of internet usage were calculated for the continuous variables, and the categorical variables were summarised with frequencies (and percentages), e.g., for sociodemographic variables, educational level, health history, and quality of life. Tests for normality and bivariate analysis using chi-square tests were conducted before the logistic regression models were developed. The binary-derived QoL (CASP-19 Score) was the dependent variable (low/high), and the variables incorporating the frequency of Internet use, and use of the Internet within the six domains, were included as the main independent variables (IV) for two sets of models.

Two different models were used in this research because the variables measuring Internet use are not independent of each other: Models 1-3a examined the association between the frequency of Internet use and QoL, and Models 1-3b examined the association between the purpose for Internet use and QoL.

Model 1a tested the unadjusted association between people’s frequency of using the internet (using a single variable) and their quality of life (QoL). Model 2a tested this association, adjusting for sociodemographic variables. The sociodemographic variables were gender, age group, educational level, wealth quintile, ethnicity, and marital status. Model 3a tested the association between the frequency of Internet use and QoL while adjusting for the sociodemographic variables and health:

Model 1a quality of life + frequency of internet use,
Model 2a quality of life + frequency of internet use + sociodemographic variables,
Model 3a quality of life + frequency of internet use + sociodemographic variables + health variable.

Model 1b tested the unadjusted association between people’s purpose for using the internet and QoL. The different domains of the purpose for internet use were represented by six variables: communication, entertainment, information access, finance management, electronic commerce and other use. Model 2b tested the association between people’s purpose for using the internet and QoL, adjusting for sociodemographic variables. Model 3b tested the association between the frequency of Internet use and QoL while adjusting for the sociodemographic variables and health:

Model 1b quality of life + Internet use purposes (six questions),
Model 2b quality of life + Internet use purposes + sociodemographic variables,
Model 3b quality of life + Internet use purposes + sociodemographic variables + health variable,

The analyses were conducted using SPSS: for the logistic regression models, the odds ratio (OR), 95% confidence intervals (CI) and significance (*p*-value) were calculated. 

## 3. Results

Table 2 shows descriptive statistics for all variables in Wave 9 *(n* = 5272). Among the sociodemographic variables, the most frequently occurring groups within the sample were people aged 60 to 69 (38.2%), women (55.2%), those from non-white ethnicity (96.1%), and those who were married (67.6%). Approximately half of the sample had completed a degree qualification or equivalent. At least one chronic disease had been diagnosed in more than half the older people within this sample. This table also shows that almost 85% of the sample used the Internet at least once a day. More than 90% of the older people used the Internet for communication, followed by accessing information (87.6%), e-commerce (77.5%), and entertainment (75.7%).

Table 3 presents the results of the chi-squared tests between the independent variables and QoL (low/high). There was a significant association between quality of life and marital status (χ^2^ = 50.453, *p* < 0.001), but not with the other sociodemographic variables (i.e., gender, ethnicity, and educational qualification). 

The chi-squared tests for trend were used to analyse the association between the quality of life and the ordinal variables. Table 3 shows that the association between QoL and each Internet use purpose was significant (*p* < 0.001), except in its association with ‘other’ purposes. Statistically significant results were also found in the relation between QoL and being diagnosed with a chronic disease (χ^2^ = 11.284, *p* < 0.001), and between QoL and the frequency of Internet use (χ^2^ = 27.504, *p* < 0.001).

Table 4 presents the logistic regression models for the relationship between frequency of Internet use, unadjusted (Model 1a), and adjusting for the sociodemographic variables (Model 2a), and, additionally, for the presence of a long-standing illness (Model 3a). There was a high degree of association between people’s Internet utilisation and quality of life (QoL), independent of the sociodemographic characteristics and the health variable. In the adjusted model, the people who used the Internet at least once a month were 85% more likely to have higher QoL (OR = 1.85; 95% CI 1.28–2.67) compared to the people who used the Internet daily. The people who used the Internet weekly were 1.45 times more likely to have higher QoL compared to those who used the internet daily (95% CI = 1.20, 1.74). 

The people aged 60–69 were less likely to have a high QoL compared to those aged 50–59 (OR = 0.77; 95% CI = 0.67, 0.89). A similar pattern was observed in the 70–79 age group. Regarding the relationship status, people who were cohabiting (OR = 1.44; 95% CI = 1.17, 1.76), or were not married (OR = 1.47; 95% CI = 1.28, 1.68), had a significantly increased likelihood of having higher QoL than those who were married.

Table 5 provides the results of the unadjusted and adjusted logistic regression models for the relationship(s) between the purpose for Internet use and QoL. The following purposes for using the Internet were significantly associated with QoL: communication, entertainment, information access, and other. The odd ratios being less than one in all three models indicate that people using the Internet for these activities were less likely to have a high QoL, compared to those who did not use the Internet for these purposes, even when adjusting for the sociodemographic variables and health conditions. Conversely, using the Internet for finance or for e-commerce was not significantly associated with QoL. The relationships between QoL and the other sociodemographic variables show similar results to those in Models 1-3a. 

## 4. Discussion

The results show a strong association between the frequency of internet use and QoL, as well as between the use of the Internet for specific purposes and QoL, even after adjusting for the sociodemographic and health-related variables. This study found that using the Internet relatively frequently (i.e., weekly or monthly) was associated with older people having lower QoL. A possible explanation for this is that increased utilisation of the Internet by older people might lead to a reduction in their physical (or social) activity, similar to previous research by Vandelanotte et al. (2009) and Kearns and Whitley (2019), who reported that Internet usage was linked to decreased physical activity. They also suggested that Internet usage had contributed to a more sedentary lifestyle, leading to several health issues [18,19].

Furthermore, the less frequently older people use the internet (e.g., less than once a day), the more time and opportunities those people have to participate in offline social activities that can add value to their life, leading to higher QoL. Prior research has shown that people who engage in more social interaction have more positive well-being [20]. This type of activity provides more available social support, which will bring better overall health. Even though this research highlights the possible benefits for older people in bridging social capital and relationships through online interaction, the Internet has been found to be more suitable for strengthening existing social connections rather than creating new ones [20]. These studies suggest that the associations between Internet use and QoL are complex.

Further, this research also shows that the higher the intensity of people using the Internet to communicate with other people, the lower their quality of life. This might happen because online communication is not as meaningful and beneficial as face-to-face communication. There might be insufficient nonverbal cues and emotions developed through online communication [21]. A survey of people having different types of work suggests that communication online, for instance by email, gives less value and intimacy than face-to-face or even telephone interaction [22]. Another explanation for these findings could be that communicating with other relations using the Internet might be associated with social isolation, at least to some extent [23]. One article supports this perspective by its findings that weekly or monthly internet use will more likely bring a better quality of life because there are more opportunities for real-world interactions and activities [24]. This will lead to lower levels of social isolation and better quality of life. 

Information access, as one of the domains of purposes for Internet use, was found to have a strong relationship with the quality of life in England’s older people. The relationship was similar to that of the frequency of Internet use, where every increase in older people’s activity in gathering information was associated with a lower chance of the people having high levels of QoL, compared to people who did not use the Internet for this purpose. Although searching online for medical information may be common among people, due to its usefulness and accessibility, some people may find that this can become excessive and potentially harmful to mental health [25]. Moreover, older adults may be particularly vulnerable to misleading information on the Internet [26]. A systematic review of pathological behaviours, such as cyberchondria, panic attacks, and other anxiety disorders, shows these results from excessive information exposure, which can strongly contribute to poor QoL [27,28,29]. Despite the negative impacts the Internet may bring to an older person’s life, several other studies have shown that gathering information via the Internet, for example, accessing news, updates on health information, travel, and leisure can also bring positive effects and opportunities to develop a personal and social connection.

Our results suggest that the quality of life in older people might not only be affected by what the people use the internet for, but also by how frequently they use the internet. Less online activity seems to bring a better quality of life for older people. However, according to the Nagelkerke R^2^ values for these models, these results only account for a small proportion of the variation in people’s quality of life. Thus, other possible factors might affect the QoL in older people that are not covered in the models presented here, and this may need further, and more in-depth, research.

Other measurements in this research were found to have a strong relationship with quality of life (QoL), such as age group, people’s relationship status, and their history of having a chronic disease. Nóbrega et al.’s study (2009) supported this, and they also found that another indicator, people’s self-perception of health, was also indicative for evaluating QoL [30]. In addition, this research shows that older people’s history of having been diagnosed with a chronic disease leads to an increased chance of achieving a higher QoL. A possible explanation for this is that people might optimise their time to explore various opportunities in life and their capabilities with a limited lifespan due to their long-term illnesses.

The relationship between marital status and life satisfaction has been reported previously [31]. This research, however, showed that, in the final adjusted model, higher QoL was associated with being unmarried. While previous research has also reported that single people had lower social loneliness compared to those in relationships [32], another study found that having commitment in a marriage can protect people from either physical, psychological, or social problems in their later life [33]. However, while being married can offer ongoing social companionship, love and support, older married people can also experience loneliness or lower well-being, for example, if they are a carer for their spouse, or if there is limited emotional interaction or intimacy. The variability in findings across studies may be methodological, e.g., because different studies are including different covariates in multivariable models [34], and/or because there may be complex interactions between marital status and other covariates that affect the association with quality of life. Further research should examine the complex relationship between marital status and QoL in older people.

Although the data were obtained from a representative sample of the population of older people in England, there was an issue with the completeness of the data: when the people with missing data were removed, the sample was reduced considerably. The reduction in data might also be due to the limitations of using self-reported questionnaires for some variables, which also may be prone to recall bias. Therefore, utilising specific objective measurements may help to prevent or minimise bias in future research. Another reason that might contribute to numerous missing values in this wave is sample attrition, which is quite common in longitudinal studies, due to mortality in older age groups, people entering residential care, or being lost to follow-up [35].

Furthermore, several waves of the ELSA Dataset have different questions and variables compared to the other waves. For instance, the data from the sample on accessing health-related information can only be found in Waves 7 and 9. Therefore, it was not feasible to undertake longitudinal research on the participants using this particular variable because of the lack of continuous information over successive waves.

The data from Wave 9 were collected in 2018-19, and it is possible the potential benefits and harms of using the Internet, and its effect on QoL, may have been attenuated during the COVID-19 pandemic. Further research should examine the association between Internet use and QoL during the pandemic, and how the relationship changed over time from before 2020.

## 5. Conclusions

This study found a strong association between older people’s quality of life and their utilisation of the Internet, even when adjusting for demographic and health-related variables. However, the relationships between the frequency of and purpose for Internet use and QoL appear to be complex. The older people who are highly active on the Internet (i.e., using it daily) have lower QoL; likewise, the people communicating more online and accessing information online tend to have lower QoL. Although some use of the Internet may be beneficial, using it too much may lead to people having less face-to-face interaction with others, and this may explain the association with lower QoL. Quality of life in older people might not only be associated with how frequently they use the internet but also with what the people use the internet for. Further research should examine how older people’s use of the Internet has changed through the COVID-19 pandemic and the effect of this on QoL.

## Figures and Tables

**Table 1:** Domains and items included in the CASP-19 [15].

Domains	Items
Control	My age prevents me from doing the things I would like to do
	I feel that what happens to me is out of my control
	I feel free to plan the future
	I feel left out of things
Autonomy	I can do the things I want to do
	Family responsibilities prevent me from doing the things I want to do
	I feel that I can please myself what I do
	My health stops me from doing the things I want to do
	Shortage of money stops me from doing things I want to do
Pleasure	I look forward to each day
	I feel that my life has meaning
	I enjoy the things that I do
	I enjoy being in the company of others
	On balance, I look back on my life with a sense of happiness
Self-realisation	I feel full of energy these days
	I choose to do things that I have never done before
	I feel satisfied with the way my life has turned out
	I feel that life is full of opportunities
	I feel that the future looks good for me
	On balance, I look back on my life with a sense of happiness

**Table 2:** Descriptive analysis of all individual variables in Wave 9 (*n* = 5272).

Measures	*n* (%)
Sex	
Male	2362 (44.8)
Female	2910 (55.2)
Age group	
50–59	1319 (25.0)
60–69	2016 (38.2)
70–79	1499 (28.4)
80–89	428 (8.1)
90 and over	10 (0.2)
Ethnicity	
White	5064 (96.1)
Non-White	208 (3.9)
Marital Status	
Married	3566 (67.6)
Cohabit	445 (8.4)
Neither/Single	1261 (23.9)
Chronic Diseases	
Yes	2770 (52.5)
No	2502 (47.5)
Education	
Degree or equivalent	2627 (49.8)
Intermediate	1148 (21.8)
No qualification	1089 (20.7)
Foreign	408 (7.7)
Wealth quintile	
Q1: lowest–140,479	1054 (20.0)
Q2: 140,480–271,668	1054 (20.0)
Q3: 271,669–423,899	1055 (20.0)
Q4: 423,900–721,099	1054 (20.0)
Q5: 721,100–highest	1055 (20.0)
Quality of Life level	
Lower QoL	2428 (46.1)
Higher QoL	2844 (53.9)
Frequency of Internet Use	
Daily	4453 (84.5)
Weekly	561 (10.6)
Monthly	141 (2.7)
Rarely (once every 3 months)	40 (0.8)
Almost never (less than every 3 months)	77 (1.5)
Internet Use	
Communication	
Yes	4923 (93.4)
No	349 (6.6)
Entertainment	
Yes	3989 (75.7)
No	1283 (24.3)
Information Access	
Yes	4619 (87.6)
No	653 (12.4)
Finance	
Yes	3315 (62.9)
No	1957 (37.1)
Electronic Commerce	
Yes	4085 (77.5)
No	1187 (22.5)
Others	
Yes	400 (7.6)
No	4872 (92.4)

**Table 3:** Bivariate analysis of the association between the quality of life of older people and the independent variables (*n* = 5272).

Measures	Value	df	Sig.
Sex ^a^	0.589	1	0.443
Age ^b^	2.859	1	0.091
Ethnicity ^a^	0.098	1	0.754
Marital status ^a^	50.453	2	<0.001 *
Education qualification ^a^	1.933	3	0.586
Wealth ^b^	0.608	1	0.435
Chronic Disease ^b^	11.284	1	<0.001 *
Frequency of Internet Use ^b^	27.504	1	<0.001 *
Internet Use ^b^			
Communication	33.606	1	<0.001 *
Entertainment	47.938	1	<0.001 *
Information Access	73.136	1	<0.001 *
Finance	19.033	1	<0.001 *
E-Commerce	24.726	1	<0.001 *
Other	1.779	1	0.182

* Significance < 0.05; ^a^ Pearson Chi-Square; ^b^ Linear-by-linear Association.

**Table 4:** Logistic regression results Model A.

Independent Variable and Overall Significance (Reference Category)	Categories	ODDS RATIO (95% CI)		
		Model 1a	Model 2a	Model 3a
Frequency of using the internet ^‡^	Weekly	1.48 (1.24–1.78) ^‡^	1.45 (1.21–1.75) ^‡^	1.45 (1.20–1.74) ^‡^
(Daily)	Monthly	2.02 (1.41–2.90) ^‡^	1.86 (1.29–2.69) ^‡^	1.85 (1.28–2.67) ^†^
	Once every 3 months	1.90 (0.98–3.69)	1.87 (0.96–3.67)	1.81 (0.93–3.55)
	Rarely	1.52 (0.95–2.41)	1.42 (0.87–2.28)	1.40 (0.87–2.25)
Sex ^(ns)^	Female		0.90 (0.803–1.00)	0.90 (0.81–1.01)
(Male)				
Age groups ^‡^	60–69		0.78 (0.68–0.90) ^‡^	0.77 (0.67–0.89) ^‡^
(50–59)	70–79		0.87 (0.75–1.01)	0.85 (0.73–0.99) *
	80–89		1.12 (0.88–1.41)	1.08 (0.86–1.37)
	90 and above		2.29 (0.48–10.95)	2.23 (0.47–10.65)
Educational Qualification ^(ns)^	Intermediate		1.05 (0.91–1.21)	1.05 (0.91–1.21)
(High qualification)	No qualification		1.05 (0.90–1.22)	1.05 (0.90–1.22)
	Foreign		0.89 (0.72–1.10)	0.89 (0.72–1.10)
Wealth quintiles ^(ns)^	Q2		0.96 (0.81–1.15)	0.96 (0.81–1.15)
(Q1: lowest–£140,479)	Q3		0.99 (0.83–1.18)	0.99 (0.83–1.18)
	Q4		1.07 (0.89–1.27)	1.07 (0.90–1.28)
	Q5		1.06 (0.88–1.27)	1.06 (0.88–1.27)
Relationship status ^‡^	Cohabit		1.44 (1.18–1.77) ^‡^	1.44 (1.17–1.76) ^‡^
(Married)	Neither/single		1.49 (1.30–1.70) ^‡^	1.47 (1.28–1.68) ^‡^
Whether has long-standing illness ^†^ (Yes)	No			0.86 (0.77–0.97) ^†^
Constant		1.09	1.14	1.24
Nagelkerke R^2^		0.01	0.03	0.03
*n*		5272	5272	5272

^‡^ Significance at the *p*-value < 0.001 level; ^†^ significance at the *p*-value < 0.01 level; * significance at the *p*-value < 0.05 level; ns = non-significant.

**Table 5:** Logistic regression results Model B.

Independent	Categories	ODDS RATIO (95% CI)		
		Model 1b	Model 2b	Model 3b
Communication_Internet Use ^†^	1 Activity	0.68 (0.53–0.88) ^†^	0.68 (0.52–0.87) ^†^	0.68 (0.52–0.88) ^†^
(No Activity)	2 Activities	0.67 (0.52–0.87) ^†^	0.66 (0.51–0.86) ^†^	0.66 (0.51–0.87) ^†^
	3 Activities	0.60 (0.43–0.84) ^†^	0.57 (0.41–0.80) ^†^	0.57 (0.41–0.79) ^‡^
Entertainment_Internet Use *	1 Activity	0.98 (0.84–1.14)	0.98 (0.84–1.15)	0.98 (0.84–1.15)
(No Activity)	2 Activities	0.79 (0.67–0.94) ^†^	0.80 (0.68–0.95) ^†^	0.80 (0.68–0.95) *
	3 Activities	0.80 (0.64–1.00) *	0.80 (0.64–1.00)	0.80 (0.64–1.01)
InformationAccess_Internet Use ^‡^	1 Activity	0.68 (0.56–0.83) ^‡^	0.69 (0.57–0.85) ^‡^	0.70 (0.57–0.85) ^‡^
(No Activity)	2 Activities	0.55 (0.45–0.67) ^‡^	0.57 (0.46–0.69) ^‡^	0.56 (0.46–0.69) ^‡^
	3 Activities	0.64 (0.46–0.87) ^†^	0.58 (0.42–0.80) ^‡^	0.58 (0.42–0.80) ^‡^
Finance_Internet Use ^(ns)^ (No Activity)	1 Activity	0.99 (0.87–1.12)	0.96 (0.84–1.10) ^‡^	0.96 (0.84–1.10)
Ecommerce_Internet Use ^(ns)^	1 Activity	0.97 (0.83–1.12)	1.01 (0.87–1.17)	1.01 (0.87–1.18)
(No Activity)	2 Activities	0.92 (0.74–1.14)	0.94 (0.75–1.17)	0.94 (0.75–1.18)
Other_Internet Use * (No Activity)	1 Activity	0.82 (0.66–1.01)	0.80 (0.65–0.99) *	0.80 (0.65–0.99) *
Sex ^(ns)^ (Male)	Female		0.89 (0.79–1.00)	0.90 (0.80–1.01)
Age groups ^‡^	60–69		0.76 (0.66–0.88) ^‡^	0.75 (0.65–0.87) ^‡^
(50–59)	70–79		0.80 (0.68–0.95) ^†^	0.78 (0.67–0.92) ^†^
	80–89		0.97 (0.76–1.24)	0.94 (0.74–1.20)
	90 and above		1.99 (0.41–9.79)	1.94 (0.39–9.51)
Educational Qualification ^(ns)^	Intermediate		1.03 (0.89–1.19)	1.03 (0.89–1.19)
(High qualification)	No qualification		1.04 (0.89–1.21)	1.04 (0.89–1.21)
	Foreign		0.87 (0.70–1.07)	0.87 (0.70–1.07)
Wealth quintiles ^(ns)^	Q2		0.94 (0.79–1.13)	0.94 (0.79–1.13)
(Q1: lowest–£140,479)	Q3		0.96 (0.81–1.15)	0.96 (0.81–1.15)
	Q4		1.06 (0.88–1.26)	1.06 (0.89–1.27)
	Q5		1.02 (0.85–1.23)	1.02 (0.85–1.23)
Relationship status ^‡^	Cohabit		1.46 (1.19–1.79) ^‡^	1.46 (1.19–1.79) ^‡^
(Married)	Neither/single		1.50 (1.31–1.72) ^‡^	1.48 (1.29–1.70) ^‡^
Whether has long-standing illness ^†^ (Yes)	No			0.86 (0.77–0.96) ^†^
Constant		3.17	3.46	3.74
Nagelkerke R^2^		0.03	0.05	0.05
*n*		5272	5272	5272

^‡^ Significance at the *p*-value < 0.001 level; ^†^ significance at the *p*-value < 0.01 level; * significance at the *p*-value < 0.05 level; ns = non-significant.

## Data Availability

Data were obtained from the English Longitudinal Study of Aging and were used with permission from the UK Data Archive, University of Essex.
